# Ultra-miniature dual-wavelength spatial frequency domain imaging for micro-endoscopy

**DOI:** 10.1117/1.JBO.29.2.026002

**Published:** 2024-02-01

**Authors:** Jane Crowley, George S. D. Gordon

**Affiliations:** University of Nottingham, Department of Electrical and Electronic Engineering, Optics and Photonics Group, Nottingham, United Kingdom

**Keywords:** spatial frequency domain imaging, miniaturization, optical properties, optical fibers

## Abstract

**Significance:**

There is a need for a cost-effective, quantitative imaging tool that can be deployed endoscopically to better detect early stage gastrointestinal cancers. Spatial frequency domain imaging (SFDI) is a low-cost imaging technique that produces near-real time, quantitative maps of absorption and reduced scattering coefficients, but most implementations are bulky and suitable only for use outside the body.

**Aim:**

We aim to develop an ultra-miniature SFDI system comprising an optical fiber array (diameter 0.125 mm) and a micro camera (1×1  mm package) to displace conventionally bulky components, in particular, the projector.

**Approach:**

First, we fabricated a prototype with an outer diameter of 3 mm, although the individual component dimensions could permit future packaging to a <1.5  mm diameter. We developed a phase-tracking algorithm to rapidly extract images with fringe projections at three equispaced phase shifts to perform SFDI demodulation.

**Results:**

To validate the performance, we first demonstrate comparable recovery of quantitative optical properties between our ultra-miniature system and a conventional bench-top SFDI system with an agreement of 15% and 6% for absorption and reduced scattering, respectively. Next, we demonstrate imaging of absorption and reduced scattering of tissue-mimicking phantoms providing enhanced contrast between simulated tissue types (healthy and tumour), done simultaneously at wavelengths of 515 and 660 nm. Using a support vector machine classifier, we estimate that sensitivity and specificity values of >90% are feasible for detecting simulated squamous cell carcinoma.

**Conclusions:**

This device shows promise as a cost-effective, quantitative imaging tool to detect variations in optical absorption and scattering as indicators of cancer.

## 1 Introduction

Gastrointestinal cancers account for one-quarter of the global cancer incidence and over one-third of all cancer related deaths.^1^ Wider population-based endoscopic screening can significantly decrease mortality,^2^ but miss rates for some types of polyps during diagnostic colonoscopies can be as high as 26%.^3^ There is therefore a need for an improved contrast imaging device that is minimally invasive and can be deployed endoscopically. To be deployed via the instrument channel of a standard colonoscope, it must have an outer diameter <3  mm, and to be suitable for population screening programs, it must be relatively low cost to manufacture and operate.

Spatial frequency domain imaging (SFDI) is a low-cost imaging technique that returns quantitative maps of absorption and reduced scattering coefficients in close to real time.^4^^,^^5^ SFDI requires a two-dimensional (2D) illumination pattern of known spatial frequency to be generated and projected onto a sample of interest, with the result being captured on a standard CMOS camera. Demodulation is then performed to obtain the high and low frequency modulation amplitudes by capturing three separate patterns equally shifted in phase ($I_{1},I_{2}$, and $I_{3}$) using the following equations:

$$I_{\mathrm{AC}}(x_{i})=\frac{\sqrt{2}}{3}{\left[{\left(I_{1}(x_{i})-I_{2}(x_{i})\right)}^{2}+{\left(I_{2}(x_{i})-I_{3}(x_{i})\right)}^{2}+{\left(I_{3}(x_{i})-I_{1}(x_{i})\right)}^{2}\right]}^{1/2},$$ (1)

$$I_{\mathrm{DC}}(x_{i})=\frac{1}{3}[I_{1}(x_{i})+I_{2}(x_{i})+I_{3}(x_{i})].$$ (2)

This is repeated on a reference material of known optical properties (and hence known diffuse reflectance values) to correct for the modulation transfer function (MTF) of the imaging system and obtain the diffuse reflectance values. Diffuse reflectance values are then used to estimate absorption and reduced scattering coefficients using a look-up table generated from either the diffusion approximation or Monte Carlo simulation as solutions to the radiative transfer equation. Obtaining the optical properties at more than one wavelength allows for the extraction of additional tissue information, such as chromophore concentration via the Beer–Lambert law. This addition of endogenous contrast information is an aid in diagnosing tissue types and has been used during breast reconstructive surgery for oxygenation imaging.^6^

Performing SFDI at more than one wavelength simultaneously is advantageous for several reasons. First, it gives the opportunity to penetrate to different depths in the sample of interest with different wavelengths.^7^ Second, it introduces the capability to obtain chromophore information, such as oxyhemoglobin and deoxyhemoglobin concentration, by measuring the variation in absorption coefficient at more than one wavelength.^8^^,^^9^ Blood oxygenation SFDI systems often operate in the red/IR, e.g., Ref. 10, but most fiber bundle systems operate in the green to avoid too much cross-coupling between fibers.^11^ Also, using just one individual wavelength (e.g., 515 nm), one can obtain different structural tissue information.

To improve the speed of SFDI systems toward real-time operation, a single phase image can be used instead of three, a technique termed single snapshot of optical properties (SSOP).^12^ SSOP uses a Fourier demodulation method to perform spatial frequency demodulation, which typically results in poorer image quality, although a method to retain more spatial resolution has been proposed using a Hilbert transform instead,^13^ and emerging convolutional neural network techniques can improve the resolution.^14^^,^^15^ SSOP has been shown to successfully quantify bowel ischaemia.^16^

SFDI has shown to return successful contrast between healthy and malignant resected esophageal and colon tissue.^17^^,^^18^ Sweer et al. imaged resected esophageal tissue from eight patients undergoing esophagectomy. By comparing regions imaged with a commercially available SFDI system from Modulim^19^ with results from histological analysis of tissue, it was determined that healthy esophageal tissue has a reduced scattering coefficient [$\mu_{s}^{\prime }=(0.73\pm 0.09)\;{\mathrm{mm}}^{-1}$] higher than the reduced scattering coefficient of both invasive squamous cell carcinoma (SCC) [$\mu_{s}^{\prime }=(0.62\pm 0.04)\;{\mathrm{mm}}^{-1}$] and Barrett’s esophagus with mild chronic inflammation [$\mu_{s}^{\prime }=(0.52\pm 0.12)\;{\mathrm{mm}}^{-1}$], in the red wavelength region ∼660  nm. The absorption coefficient of healthy esophageal tissue ($\mu_{a}=(0.038\pm 0.009)\;{\mathrm{mm}}^{-1}$] is lower than invasive SCC [$\mu_{a}=(0.077\pm 0.009)\;{\mathrm{mm}}^{-1}$] and comparable to Barrett’s esophagus with mild chronic inflammation [$\mu_{a}=(0.029\pm 0.009){\mathrm{mm}}^{-1}$], at a wavelength of ∼660  nm. Nandy et al. found that healthy colon tissue has a higher reduced scattering coefficient than malignant colon tissue and a lower absorption coefficient across the wavelength range of 460 to 630 nm.

SFDI is an attractive choice for an imaging modality because it does not require high-powered lasers, sensitive detectors (mobile phone cameras are sufficient), or complex optical components. It is therefore relatively low cost to manufacture and operate devices, and they can be miniaturized easily. As a result, a number of SFDI systems exist; these include large commercial systems,^19^ portable handheld systems,^20^ handheld 3D-printed systems,^21^ and compact multispectral imaging systems.^22^ However, in most existing systems, the projector element remains costly and difficult to miniaturize, being typically composed of either a digital micromirror device (DMD) projector,^20^ a motorized grating,^21^^,^^22^ or a static spatial frequency transparency.^23^

There have been a number of approaches to miniaturize SFDI projectors to make them suitable for endoscopic deployment. Fixed gratings have been used to achieve SSOP via rigid endoscopes.^24^ Although SSOP is advantageous as it reduces acquisition times, it poses several disadvantages, such as reduced image quality due to the use of filtering a single image. The previously developed probe is rigid in nature and not suitable for imaging in the gastrointestinal tract. Fixed gratings have also been used for optical sectioning via flexible imaging fiber bundles.^25^ The use of a micro camera is advantageous over imaging through a fiber bundle as an imaging fiber bundle is highly sensitive to vibrations, cross coupling, and fiber movements, making the reconstruction of images challenging. Phase-shifted illumination has been demonstrated via an imaging fiber bundle,^26^ but the use of DMDs is relatively high cost, and commercial fiber bundles projection only support high fidelity fringes at green wavelengths due to increased cross-coupling between cores in red wavelengths.^11^ Ultra-thin fiber arrays have been used to create fringe patterns interferometrically for profilometry but not, to our knowledge, for SFDI.^27^

None of these existing systems are suitable for routine endoscopic deployment in the gastrointestinal tract because they either use DMD-based projectors, which are costly and cannot be sufficiently miniaturized; use fiber bundles, which produce low-quality fringe patterns at a limited set of wavelengths and only record low resolution images; or use rigid endoscopes, which are not flexible enough.

We have therefore developed an ultra-miniature SFDI system, with an outer diameter 3 mm that uses a fiber array to interferometrically produce fringe patterns at green (515 nm) and red (660 nm) wavelengths and records images at 320×320  pixel resolution using a micro camera. The prototype packaging is sufficiently small that is it compatible with the instrument channel of a standard colonoscope. This makes the device comparable to the thinnest previous SFDI system designs that used fiber bundles to achieve a total diameter of 2.7 mm.^25^

We first compare optical property measurements in our ultra-miniature system to that of a conventional bench-top system and find agreement between absorption and reduced scattering coefficients of 15% and 6%, respectively. We show the potential to operate the system at more than one wavelength simultaneously, enabling rapid tissue property measurements. This device therefore shows potential to be deployed endoscopically for *in-vivo* gastrointestinal imaging to detect optical properties as potential indicators of cancer.

## 2 Methods

### 2.1 Component Design and Selection

The primary components needed for an SFDI system are a source of pattern projection and an image detector to capture the projected patterns on a sample of interest. We chose to use an optical fiber array as the source of projection patterns and a micro camera as the detector.

**Fig. 1:**
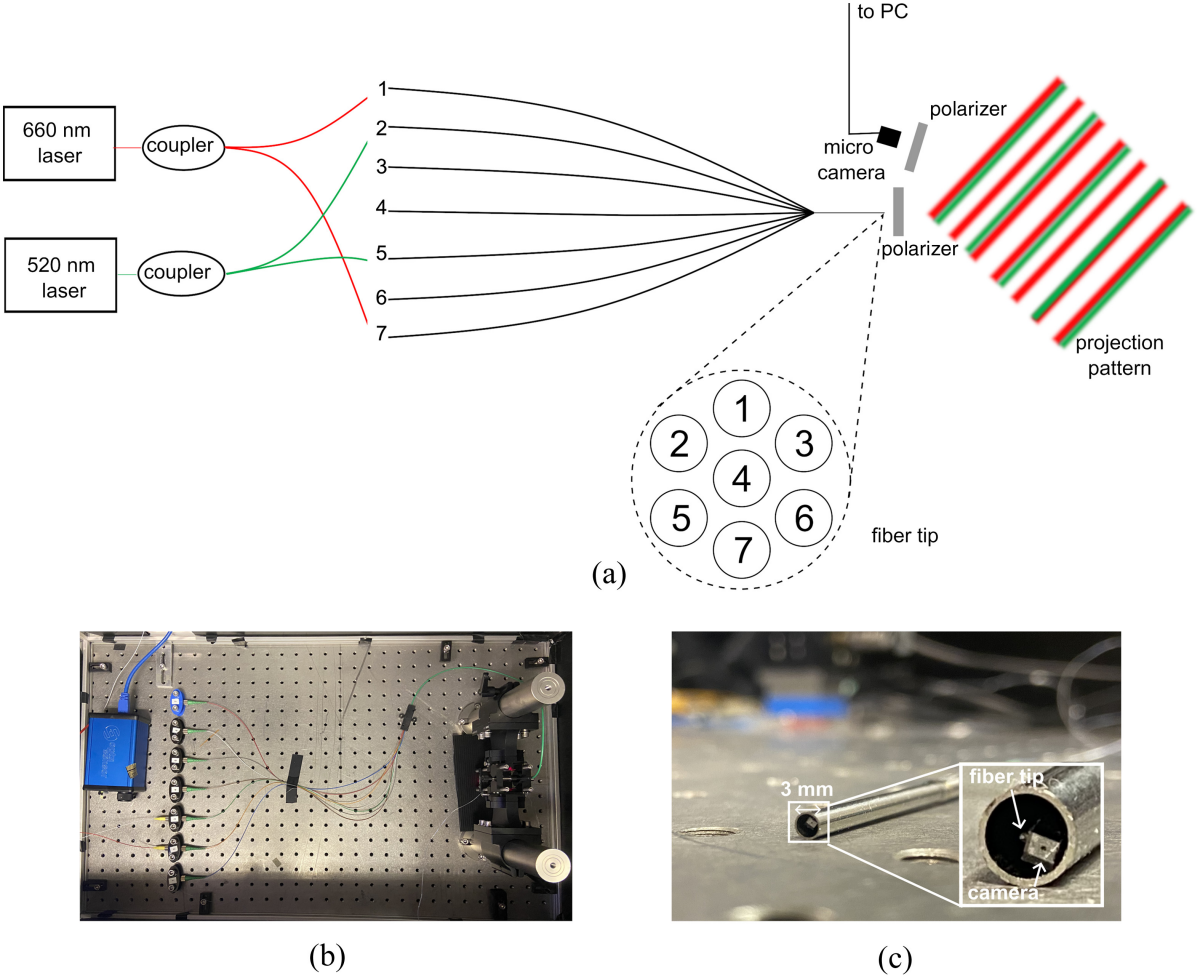
Proposed ultra-miniature SFDI system. (a) Schematic of fiber array in ultra-miniature SFDI system showing dual wavelength illumination simultaneously. Light passes from the two lasers into the fiber array via a selection of seven single-mode fiber input ports. At the tip of the fused taper, the fibers are spaced in a hexagonal array, providing three possible spacings. Crossed polarizers are placed in front of the fiber tip and the micro camera to reduce specular reflections from the imaging sample. (b) Photograph of the experimental setup. (c) Prototype device package of 3 mm diameter with inset showing zoomed in view of fiber tip and camera.

**Fig. 2:**
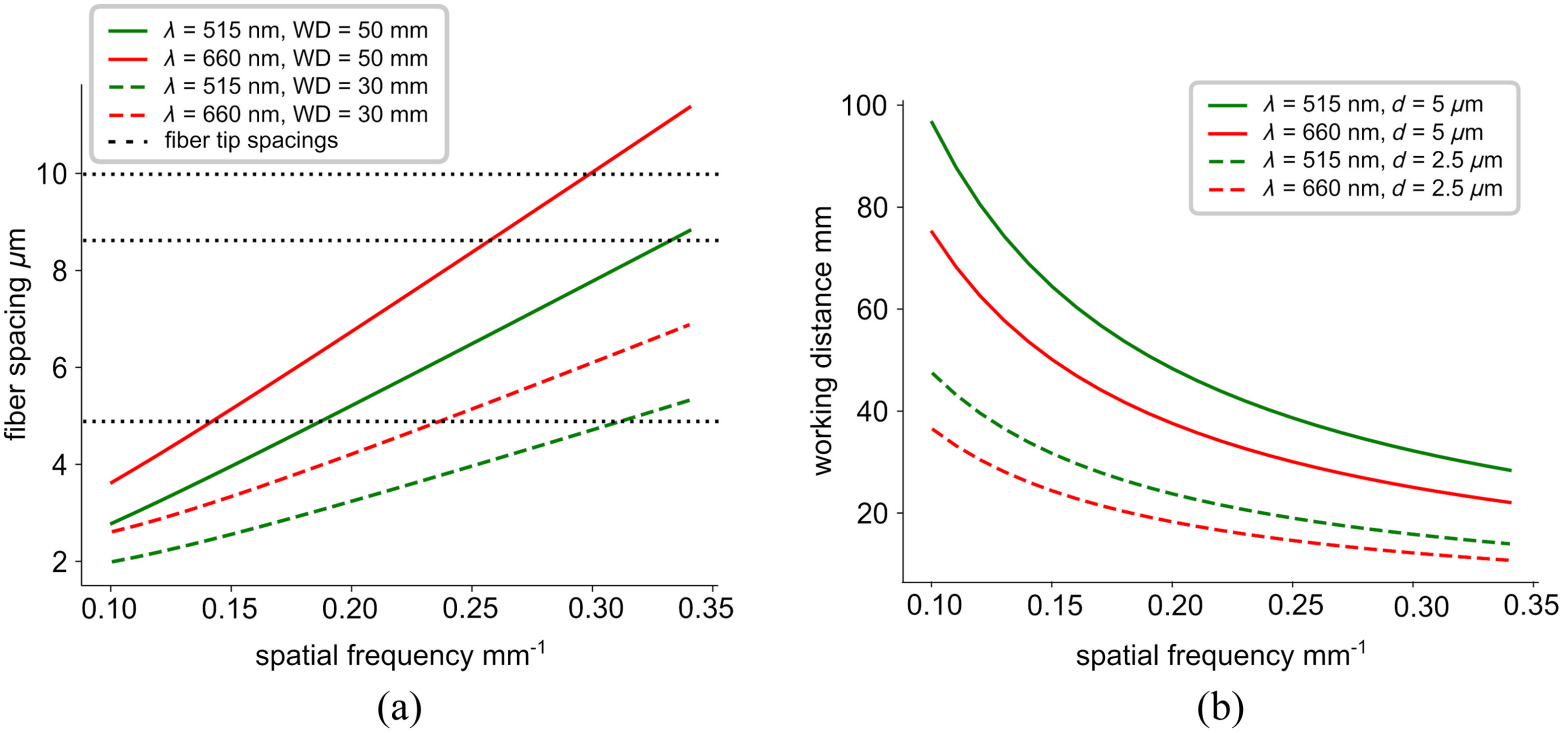
Determination of the desired fiber spacing to produce spatial frequencies within our range of interest of 0.1 to $0.3\;{\mathrm{mm}}^{-1}$. (a) Addressable spatial frequency projection at WD of 50 mm (solid lines) and 30 mm (dashed lines). The dotted lines represent the three possible fiber tip spacings of 5, 8.66, and 10  μm. (b) Proposed design of the spatial frequency projection at various WDs for fiber tip spacing (d) of 5  μm (solid lines) and 2.5  μm (dashed lines), useful for smaller WDs.

To create an ultra-miniature fringe projector without using DMD elements, we designed a customized 2D pitch-reducing optical fiber array (PROFA™, Chiral Photonics, New Jersey) to create fringes interferometrically, shown in Fig. 1. The fiber array was designed to produce interference patterns within a widely used spatial frequency range (0.1 to $0.3\;{\mathrm{mm}}^{-1}$^28^^,^^29^) at an initial test working distance (WD) of 50 mm when two adjacent channels are illuminated by the same laser source. To compute the required fiber spacings, we used a double slit equation:

mλ=d sin θ, (3)

where m is the number of the interference line spacings from the central point, λ is the wavelength of light, d is the distance between slits, and θ is the angle of projection. The desired wavelength was chosen to be 660 nm. The distance from slit to projection pattern, i.e., the WD, was chosen initially to be 50 mm, which is the maximum WD of the camera. Using Eq. (3), we can therefore determine the spacing d required to produce our spatial frequencies of interest. The fabricated fiber array has spacings of 5, 8.66, and 10  μm, which produces spatial frequencies of 0.15, 0.25, $0.3\;{\mathrm{mm}}^{-1}$ at 660 nm, as shown in Fig. 2(a). We can then determine the spatial frequency projection at varying fiber-to-sample WDs, shown in Fig. 2(b). Typical endoscope WDs are 20 to 30 mm,^30^ which is achievable using the 5  μm spacing option of our array with a $0.3\;{\mathrm{mm}}^{-1}$ spatial frequency, though in future designs, a 2.5  μm spacing could enable even shorter WDs. The seven fiber channels are spaced at the tip as shown in Fig. 1. The light sources used are a 5 mW 660 nm laser diode (LPS-660-FC, Thorlabs) and a 3 mW 515 nm laser (LP515-SF3, Thorlabs).

The camera chosen is a 1×1  mm micro camera module (Osiris M module, OptaSensor, Germany). The camera has a resolution of 320×320  pixels, with an individual pixel size of 2.4  μm. An in-built lens placed in front of the sensor provides horizontal and diagonal fields of view of 68 deg and 90 deg, respectively, accompanied by a depth of focus of 5 to 50 mm. The camera module produces a 12 bit RGB raw image output. The camera is accompanied by software to control camera parameters, such as exposure, gamma correction, and black level correction. The automatic exposure correction was disabled so that all image frames contain the same optical power ranges. The micro camera has a frame rate of up to 50 fps, but here we typically operate it at 10 fps due to capture software limitations. However, 10 fps is the minimum rate required for proper endoscopic visualization.^31^

To minimize specular reflections present on the imaging sample, adhesive-backed polymer polarizer sheets are placed in front of the camera and fiber tip to create cross-polarization. The camera is also placed at a small angle of 4 deg to the fiber to further limit specular reflections on the imaging sample. This angle is smaller than conventional SFDI systems,^6^ but it is more amenable to miniaturization. Previous work has shown that this angle can still produce high quality optical property maps^32^

### 2.2 Phase-Tracking Algorithm

An inherent property of an interferometer such as our fiber array is that the sinusoidal pattern produced shifts over time due to mechanical drifts, vibrations, temperature, and intensity variations.^33^ Conventional wisdom may suggest using a complex setup consisting of a phase-shifting control system and a piezoelectric transducer driver to stabilize and control this phase shift.^27^ However, we exploit the natural phase drift to our advantage via a phase-tracking algorithm.

**Fig. 3:**
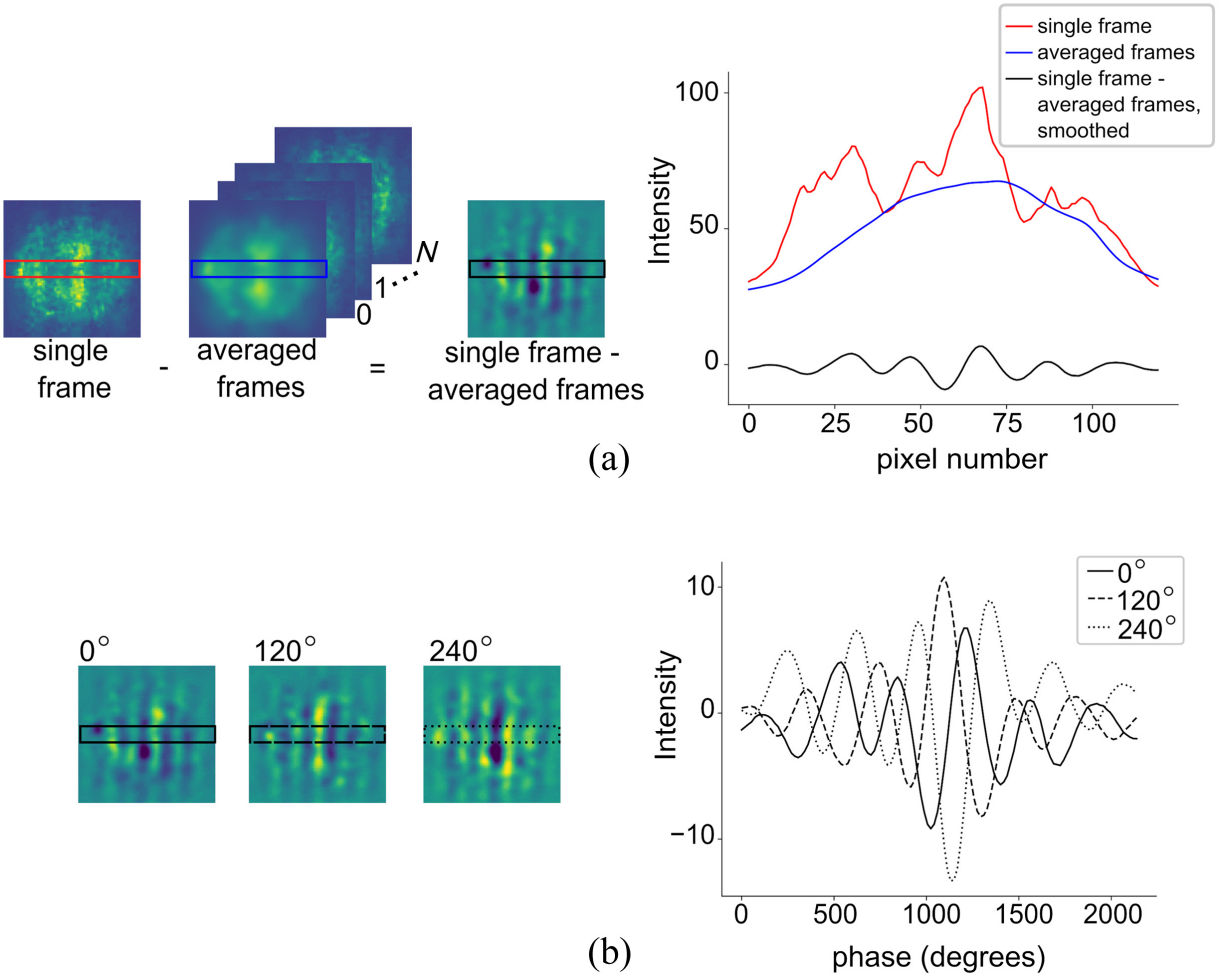
Characterization of fringes and phase tracking. (a) Image of selected zeroth frame and average of all frames, where N is the total number of all frames within the video capture, and corresponding cross sections. The single frame-averaged frames had a smoothing filter applied. (b) Image of zeroth frame, 120 deg shifted frame, and 240 deg shifted frame and corresponding cross sections, all with smoothing filters applied.

A video, typically 10 to 20 s, is first recorded of the shifting sinusoidal pattern on a sample of interest. This timeframe is selected purely for experimental convenience, but in practice, only the first second or so of the recorded frames is required. To determine which frames to use for demodulation, we first take an average of all frames within the video and subtract this from each individual frame. This allows us to visualize the spatial frequency pattern with reduced noise [see Fig. 3(a)].

We then take an average across several rows within the frame, applying a smoothing filter, and plot the sinusoidal pattern. We select a zeroth frame and designate the phase of the extracted sinusoid to be 0 deg. Next, we calculate the average distance between the adjacent maxima of this sinusoid in pixels. This value gives the period of the pattern, in pixel units. Custom Python code then cycles through all frames in the captured video and selects frames of equal intensity variation with sinusoidal projections that have relative phases of (120±10)  deg and (240±10)  deg from the selected zeroth frame [see Fig. 3(b)]. Frames in which the sine wave is non-discernible or the intensity variation between peak and trough is low relative to the zeroth frame are disregarded. This eliminates frames in which the coherence is temporarily disturbed while perturbations are still in progress. We then select these frame numbers from the initial video and demodulate the images using Eqs. (1) and (2).

### 2.3 Imaging Homogeneous Tissue-Mimicking Phantoms

To perform initial validation of the system, we fabricated tissue mimicking co-polymer in oil phantoms with tunable optical properties by controlling concentrations of ${\mathrm{TiO}}_{2}$ and Nigrosin dye.^34^ The fabricated phantoms had a thickness of 30 mm and were ensured to be non-transparent to meet the semi-infinite thickness requirement of SFDI.^35^ We fabricated two phantom batches: one with increasing amounts of dye stock solution from 0.5 to 1 g (0.0015 to 0.0030 w/v%) corresponding to an absorption coefficient range of 0.006 to $0.017\;{\mathrm{mm}}^{-1}$ at 660 nm and the second with increasing amounts of ${\mathrm{TiO}}_{2}$ from 0.07 to 0.13 g (0.07 to 0.13 w/v%) corresponding to a reduced scattering coefficient range of 0.52 to $0.99\;{\mathrm{mm}}^{-1}$ at 660 nm. The batches with an increasing dye stock solution each had 0.1 g of ${\mathrm{TiO}}_{2}$ and the batches with an increasing ${\mathrm{TiO}}_{2}$ each had 0.5 g of the dye stock solution to ensure the semi-infinite material requirement was met. We chose these optical property ranges as they are within the optical properties of interest of typical gastrointestinal tissue samples^17^ and they had previously been calibrated in the literature using a double integrating sphere (DIS).^34^

For the purposes of comparison, we imaged phantoms in both our ultra-miniature system and a standard bench-top SFDI system. The bench-top system was built in-house using a commercial projector (LG Minibeam PH150g HD ready mini projector), a Raspberry Pi camera, crossed polarizers placed in front of the camera and projector, and a 635 nm filter placed in front of the camera to ensure that only red light was captured. The system was validated against phantoms with optical properties measured in a DIS system and found to be in agreement with a margin of error around 20%, which is within the typical expected range.^36^

The phantoms were placed in the bench-top system such that the projector is placed at a 6 deg angle to the plane of the camera, the projector–camera distance is fixed at 35 mm, and the distance from the projector–camera plane to the base breadboard is 220 mm. The diffusion approximation was used to generate a look-up table to recover the optical properties of the phantoms. Because our ultra-miniature system uses a laser at 660 nm and the bench-top system uses white-light source with a camera filter at 635 nm, a small adjustment for wavelength is required. First, separate LUTs are computed for each of the wavelengths. Second, an adjustment based on multiwavelength measurements from a DIS system is applied.^34^ For these particular phantoms, the measured difference via DIS between absorption and reduced scattering coefficients from 635 to 660 nm is 14% and 3%, respectively. Therefore, we adjust reference optical properties for optical property calculation depending on whether we were using the bench-top or ultra-miniature system. When imaging phantoms at 515 nm in the ultra-miniature system, the LUT and reference optical properties were also adjusted accordingly.

We placed the phantoms such that the top of the phantom was 50 mm from the distal end of the imaging probe and the projection pattern was in the center of the sample. We took videos of the shifting projection pattern on the phantom for 10 to 20 s. The video was then input to Python phase-tracking code for processing, described in Sec. 2.2, to find the exact frames needed to calculate the optical properties. We imaged each phantom at three different spatial frequencies by illuminating three different fiber channel combinations. We calculated the optical properties using a look-up table generated from the diffusion approximation. For each phantom, we calculated the optical property maps a total of 18 times, using every other phantom as a reference in turn for each spatial frequency. This approach helps to average out errors arising from mismatches in expected optical properties of phantoms, which arises in turn due to discrepancies between DIS and SFDI measurements, which can be up to 20%.^36^ Finally, the mean of all 18 optical property maps is used to determine the absorption and reduced scattering coefficients. A 2D Gaussian filter with a standard deviation of 5 pixels was applied to the resultant optical property maps using scipy.ndimage.gaussian_filter.

### 2.4 Dual Wavelength Imaging

Multi-wavelength imaging is possible with this system as the fiber array consists of seven channels and only two are needed per wavelength to produce an interference pattern. Therefore, this system has the potential to explore up to tri-wavelength simultaneous illumination. We imaged three phantoms with 660 nm projection only, then 515 nm projection only, and finally with 660 and 515 nm projected simultaneously. We performed dual-wavelength imaging by illuminating channels 1 and 7 with 660 nm and channels 2 and 5 with 515 nm, producing spatial frequency patterns of 0.3 and $0.2\;{\mathrm{mm}}^{-1}$, respectively, at a 50 mm WD. A video was captured of both illumination patterns simultaneously, and the analysis was carried out by extracting the red and green channels from the video capture. Following the same process in Sec. 2.2, the fringes of interest were selected and optical properties were calculated. Expanding the existing system to three wavelengths (tri-color) would be possible by connecting an additional blue laser (e.g. 450 nm) to two unused illumination channels and extracting the blue channel of the captured video. Multi-wavelength imaging would probe different depths and could be used to image optical properties of layered material.

### 2.5 Extrapolating Performance to Clinical Applications

The ultra-small size of our prototype device may incur performance penalties compared to standard bench-top SFDI. Qualitatively, this appears as noise in measured images. However, the ultimate goal of the tool is to provide sufficient contrast for identifying diseased tissue compared to healthy tissue. To assess this, we post-process our optical property measurements to estimate the sensitivity and specificity that could be achieved in a clinical device. This is then compared against American Society of Gastrointestinal Endoscopy (ASGE) guidelines, which require a new device to demonstrate 90% sensitivity and 80% specificity to be used *in lieu* of biopsy sampling.^37^

To extrapolate our device’s potential clinical performance, we simulate random measurements based on previously measured absorption and scattering statistics of healthy esophageal tissue, Barrett’s esophagus, and SCC.^17^ Because of the non-negativity and underlying multiplicative nature of absorption and scattering processes, a log-normal distribution is used to generate samples.^38^ A baseline model that estimates that performance under the assumption that intra-sample variation is purely biological in origin is first created. To adapt this into the model used here, we add an additional term to the variance based on error measurements observed from our ultra-miniature system.

500 random training samples are generated for the healthy, Barrett’s, and SCC tissue types, and two binary linear support vector machine (SVM) classifiers are trained (healthy versus Barrett’s and healthy versus SCC). The SVMs are implemented using the MATLAB software package. A further 500 validation samples are then generated for each of the three classes and passed through the trained classifiers. Based on the classification error rates, sensitivity and specificity for the baseline and ultra-miniature system are estimated.

Another important clinically relevant parameter is the minimum size lesion that can be detected: lesions smaller than 6 mm are not typically considered high risk and thus are not removed. There are two aspects to this: the first is the effect of the image elements and apertures on the MTF, which can be estimated by imaging a resolution test chart. The second aspect is the impact of noise in the recovered optical properties. One way of considering this is that increased noise increases the uncertainty when identifying the “edge” of a step function. This in turn translates to a larger range of spot sizes, reducing the resolution. To quantify this, we first image two phantoms with different optical properties placed side by side so they are touching, which should ideally produce a “step” function. This step function manifests in our images as a sigmoid due to the system MTF. We then use a bootstrap sampling approach to generate random sigmoid functions and compute their probability of fit to step at the boundary between the phantoms using the measured noise statistics as the parameters of Gaussian probability density functions. We then reject samples according to their probability (i.e., their likelihood of being correct) to obtain a representative sample of likely sigmoid functions that could adequately fit the boundary between phantoms. Next, these sigmoids are differentiated to give their corresponding impulse function (i.e., spot function for imaging). By taking the mean across these sampled spots and computing the full-width half maximum, we get an estimate of how the expected ability to resolve structures changes as a result of noise.

## 3 Results

### 3.1 Projector Performance

The expected spatial frequency of the projected illumination pattern is comparable to the desired spatial frequency with 12% and 7% error for 660 and 515 nm, respectively. Some channels produce clearer interference patterns than others due to cross talk between fibers. This also results in the interference pattern from some channels being more stable than others in time, with interference patterns being stable for <1  s under typical operating conditions, but >10  s if the fibers are kept still.

**Fig. 4:**
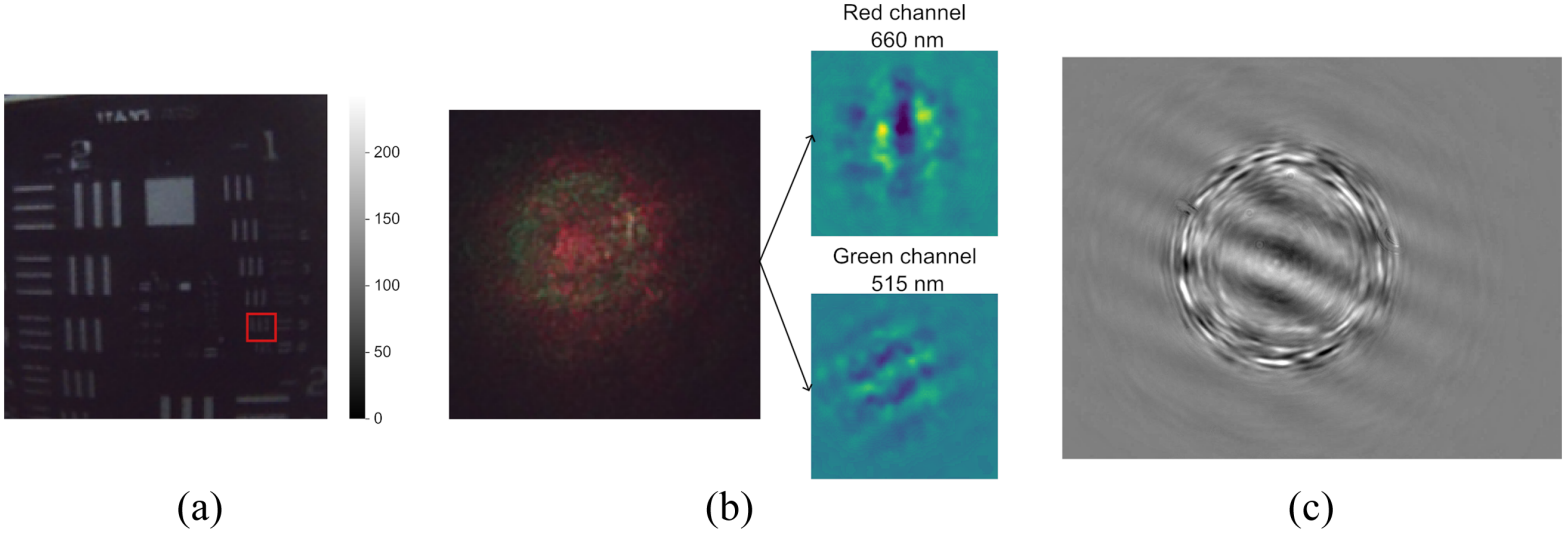
Raw performance of the camera and projector. (a) Image of USAF target captured with a mini camera module taken with room lights on; (b) image captured with mini camera module of dual wavelength projection from fiber tip showing extracted red and green channels, respectively; and (c) direct capture on image sensor of projected fringe pattern, showing good fringe contrast within a region of circular interference.

Through imaging a resolution target (R3L3S1N - Negative 1951 USAF Test Target, 3” x 3”, Thorlabs, United Kingdom), we determined the resolution of the imaging system to be 0.793  lp/mm at a WD of 50 mm, shown in Fig. 4(a). Figure 4(b) shows the raw performance of the projection fiber with green and red wavelengths onto an absorbing and scattering phantom. This represents a typical raw image required from the camera. Finally, Fig. 4(c) shows a direct capture on a CMOS image sensor of the projected pattern from the fiber. This shows that clear fringes are produced within an envelope, but that there is also some noise arising from aperture and cross-talk effects forming a ring around the target area. We, therefore, restrict our analysis to regions within this ring where fringes are projected with high quality.

### 3.2 Phase-Tracking Stability

**Fig. 5:**
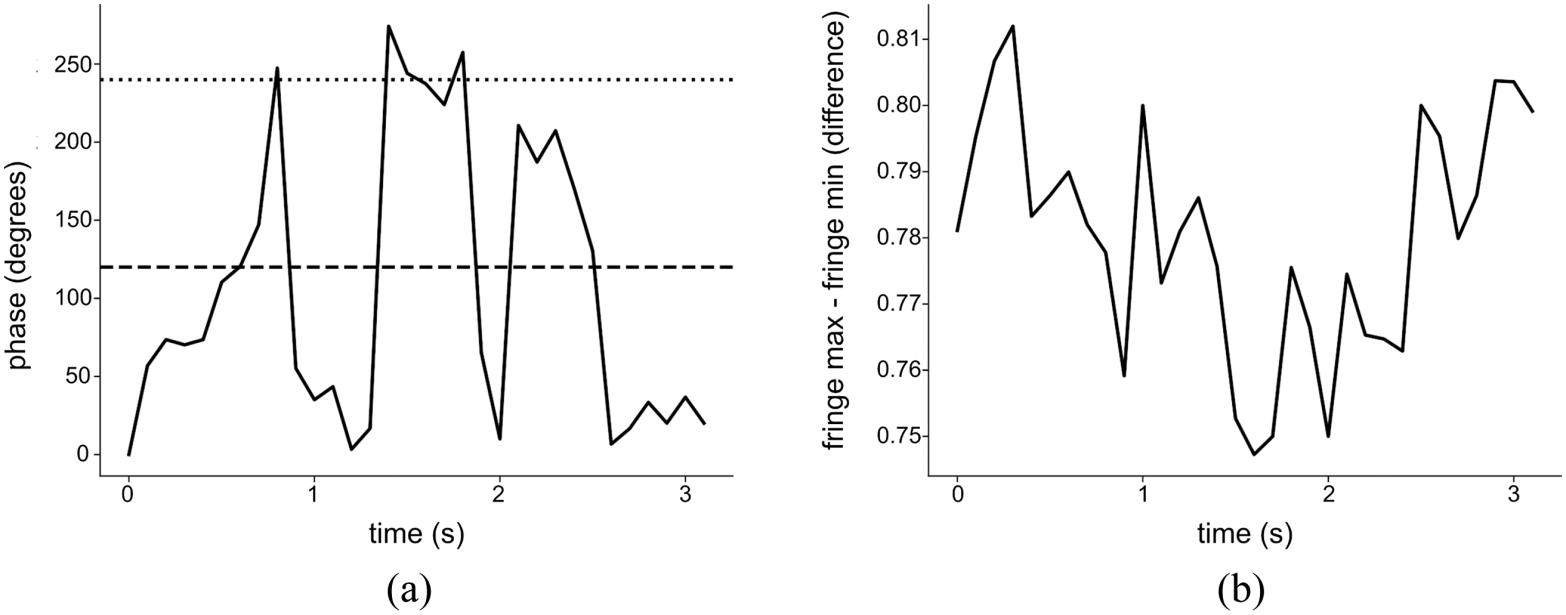
Fiber array performance. (a) Phase of frames versus time and (b) fringe contrast, defined as the difference from maxima to minima of interference fringes detected versus time.

We analyzed the fringe phase shift and contrast for 15 recorded videos, during which we continually perturbed the fiber by hand to simulate realistic usage. An example trace of fringe phase versus time is shown in Fig. 5(a), which indicates that the required three phases can be obtained under 1 s. Figure 5(b) shows the calculated difference from maximum to minimum of the interference fringes as a function of time, i.e., the fringe contrast. The contrast appears relatively stable over the 3 s time interval. To further analyze the combined effect, we used Poisson statistics across these 15 captures to estimate the expected time at the maximum frame rate required to obtain three usable frames (defined as having sufficiently high contrast across three phases) with 99% probability. We found that this varied from 0.42 to 2.34 s with a mean of 1.17 s. This may be sufficient for practical imaging provided the probe can be held within the same field of view for 1 to 2 s.

To examine the stability of the fringe envelope, we averaged all frames together for individual video captures and plotted the cross-sectional envelope profile. By repeating this across five separate video captures using a range of different phantoms, we observed how the fringe envelope varies. Given that the envelope shape is largely determined by the fiber exit aperture, we would expect the shape to be relatively uniform across different samples, although effects such as cross-talk between fibers may cause a variation. In addition, fluctuations in laser power, laser polarization state, or small transients in relative power in different fibers due to manual perturbations may influence the envelope.

**Fig. 6:**
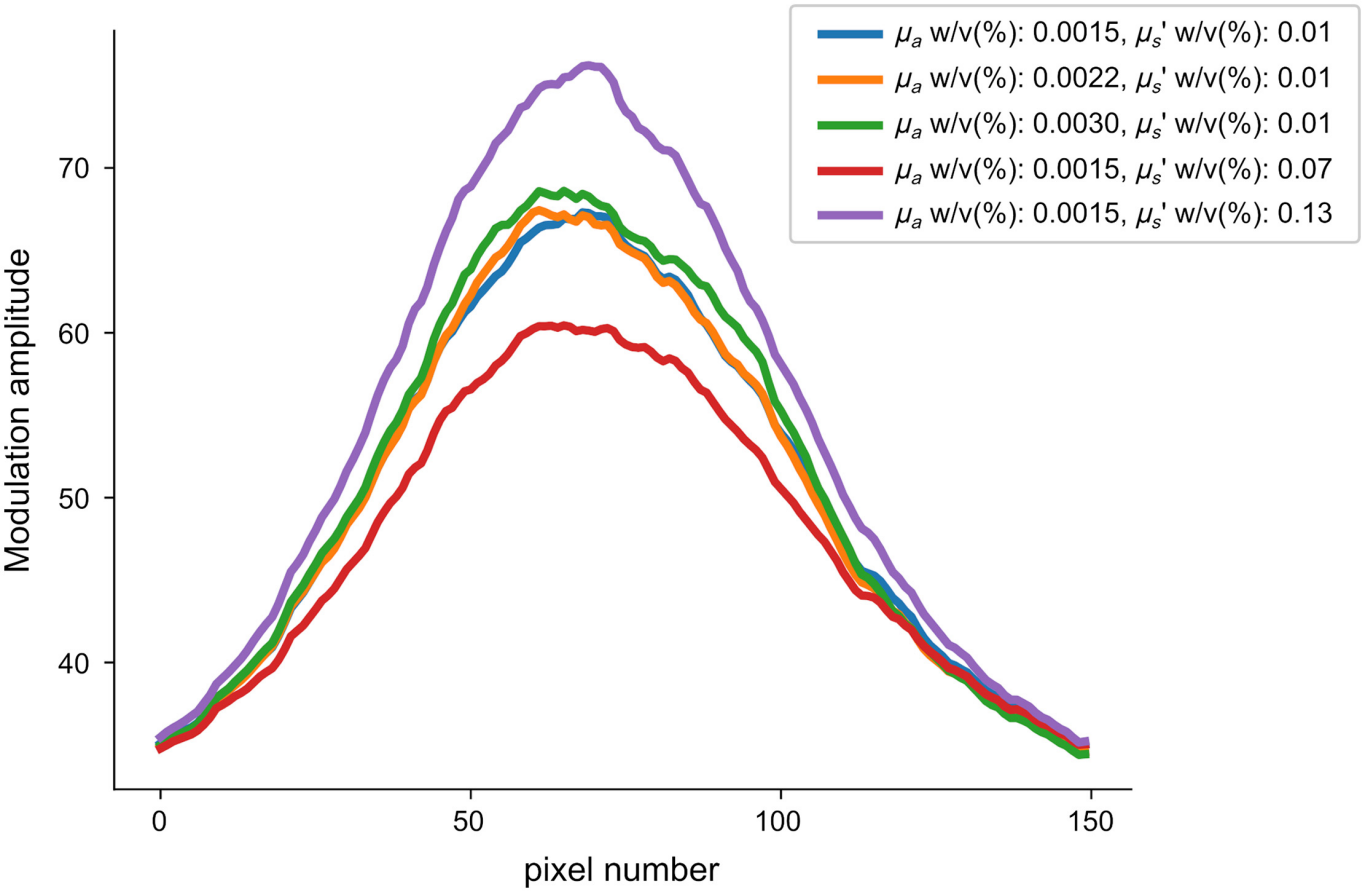
Plot of average fringe envelope for five different captures using phantoms with different optical properties. Though the amplitude of the envelop varies in intensity, the position and shape stay relatively constant.

From our results, shown in Fig. 6, we observe that the shape of the envelope is fairly consistent across different captures, though the absolute scale does appear to change within a range of ±10%. In future systems, this could be partially compensated by having a power meter recording laser output that can be used later for normalization.

We therefore expect that there may be some variation between the reference phantom envelope and the measurement: the envelope will be approximately correct but the amplitudes may vary, which could be a contributing source to the overall system error, in the region of 10%.

### 3.3 Comparing Ultra-Miniature SFDI System to Bench-Top SFDI System

**Fig. 7:**
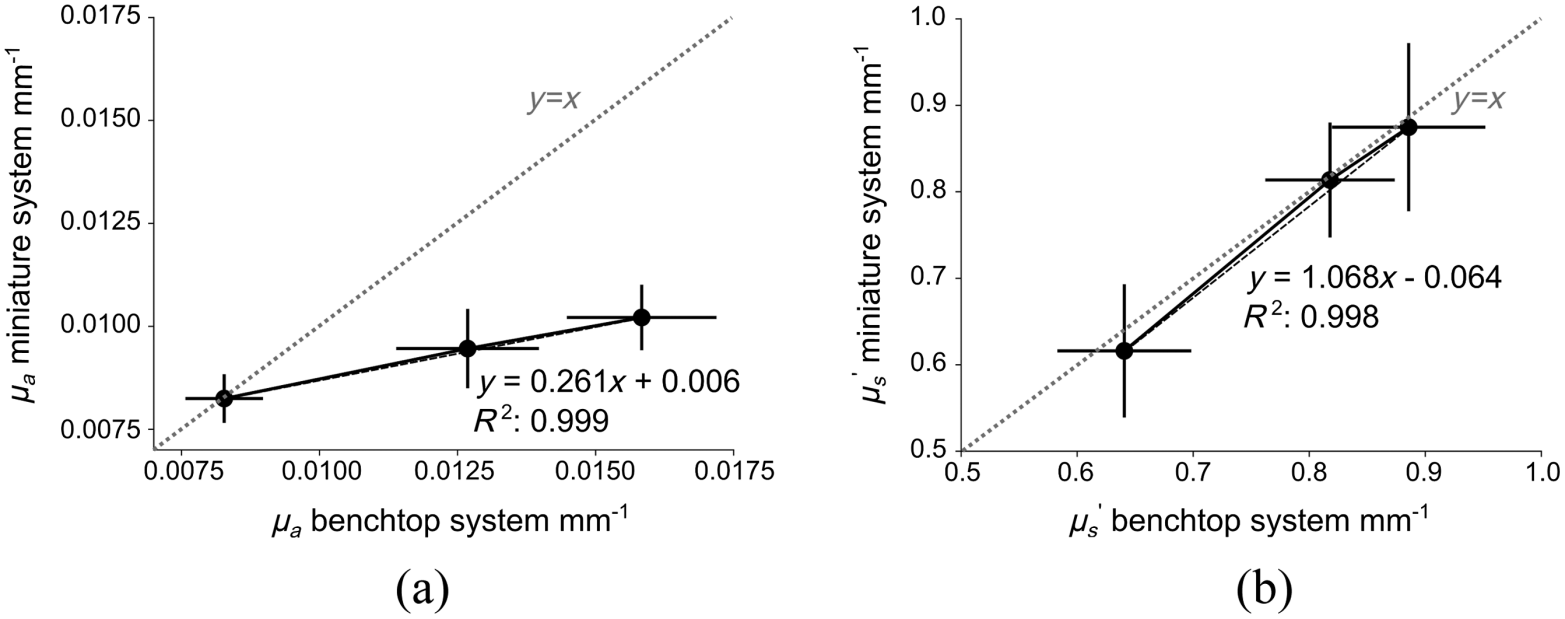
Comparison of the bench-top SFDI system and the ultra-miniature system: (a) absorption coefficient and (b) reduced scattering coefficient measured from bench-top system (x axis) and ultra-miniature system (y axis). Error bars represent the standard deviation across the image.

We then compared optical property measurements from our conventional bench-top SFDI system to our ultra-miniature SFDI system. The results are shown in Figs. 7(a) and 7(b). We found that the average standard error in absorption and reduced scattering coefficients between the ultra-miniature system and the bench-top system were 15% and 6%, respectively. However, we note that the slope of the absorption graph is much less than unity, reaching as high as 60% in the high absorption range. This may be due to a range of factors including stray background light, non-ideal sinusoid shapes, and the resulting mismatch in the LUT or varying envelope amplitudes. Because the absorption is consistently underestimated, this may be rectified using additional calibration steps, for example, phantom-based LUT generation^39^ or realistic simulation of the fiber imaging setup.^32^

### 3.4 Imaging Typical Gastrointestinal Conditions with Ultra-Miniature SFDI System

**Fig. 8:**
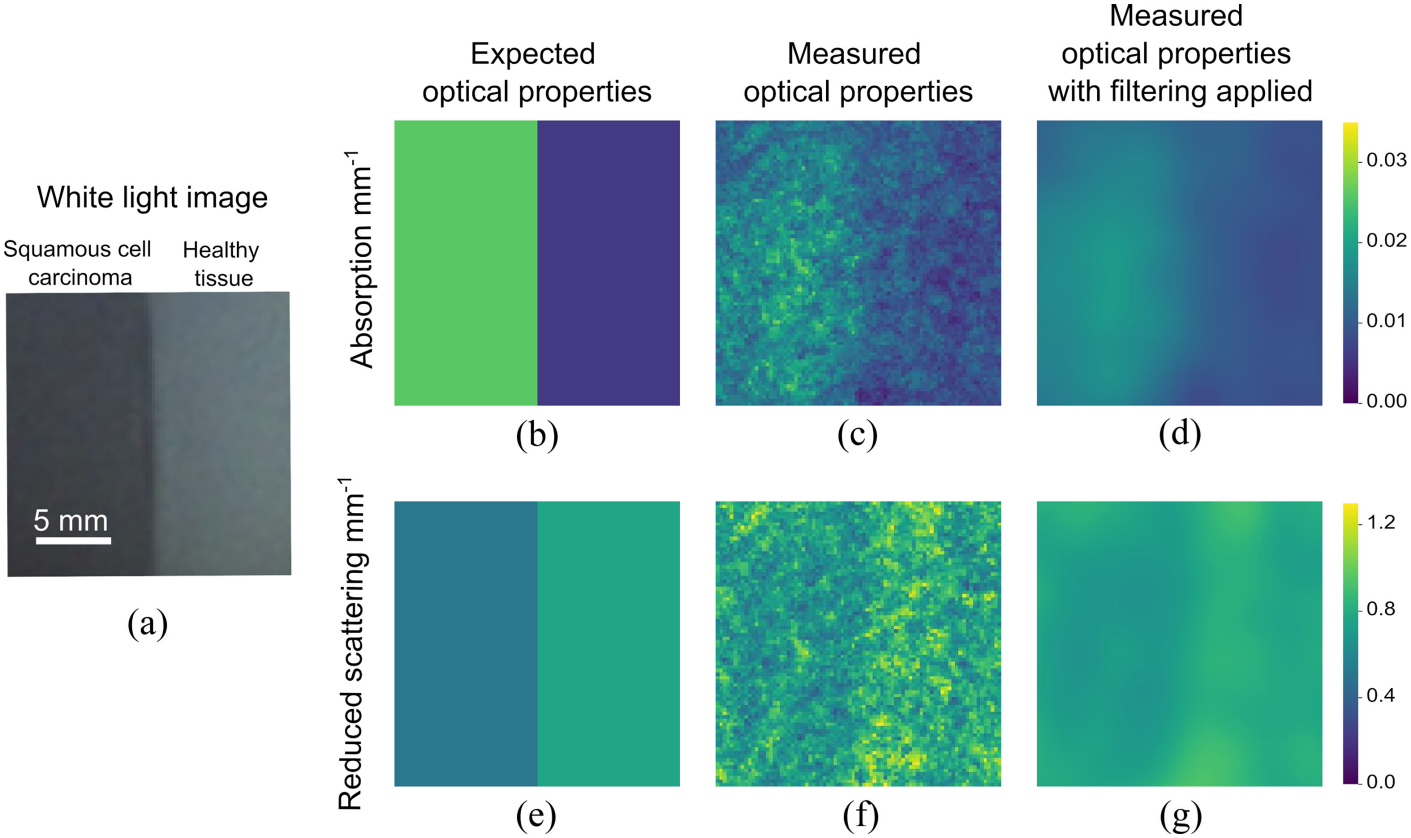
Imaging a phantom simulating esophageal tissue at 660 nm: (a) white light image of two phantoms with different optical properties side by side; (b) expected and (c) measured absorption coefficient of phantoms; (d) measured absorption coefficient with smoothing filter applied; (e) expected and (f) measured reduced scattering coefficient of phantoms; (g) measured reduced scattering coefficient with smoothing filter applied. Expected optical properties are computed from the mean measured values of the individual phantoms measured in the bench-top SFDI system.

We fabricated two phantoms to simulate gastrointestinal tissue states: one with optical properties mimicking SCC and the second with optical properties mimicking healthy esophageal tissue, and we placed them side by side. SFDI imaging of this sample was then performed at 660 nm, with the resulting optical property maps shown in Figs. 8(c) and 8(f), and resultant optical property maps with filtering applied shown in Figs. 8(d) and 8(g).

**Fig. 9:**
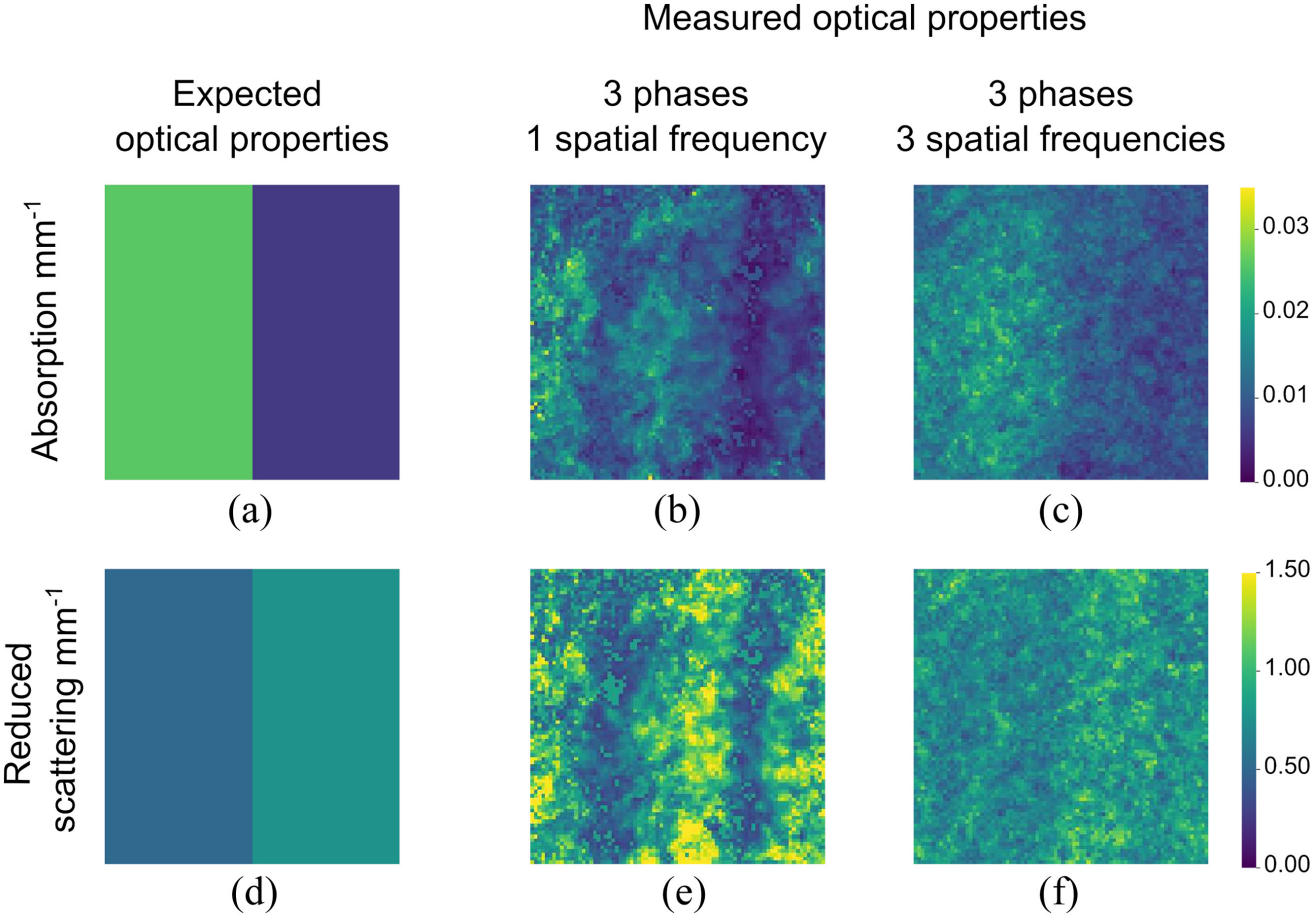
Visual representation of averaging over several spatial frequencies to reduce speckle noise: (a) expected absorption map; (b) recovered absorption map using a single spatial frequency; (c) recovered absorption map by averaging three different spatial frequencies (i.e., different fibre pairs); (d) expected scattering map; (e) recovered scattering map using a single spatial frequency; and (f) recovered scattering map by averaging three different spatial frequencies.

To illustrate the effect of speckle averaging, we compared the optical properties of the same phantoms measured with just a single spatial frequency to three spatial frequencies averaged together. The results are shown in Fig. 9: Figs. 9(a) and 9(d) represent the expected optical properties of the phantoms, Figs. 9(b) and 9(e) show the optical properties measured from a single spatial frequency projection, and Figs. 9(c) and 9(f) show the optical properties measured by taking the average over three different spatial frequency projections. The standard deviation of pixels in the absorption coefficient maps reduces from 0.0049 to 0.0044 in Figs. 9(b) and 9(c). The standard deviation of pixels in the reduced scattering coefficient maps reduces from 0.32 to 0.15 in Figs. 9(e) and 9(f).

Using our post-processing simulation with SVM classifiers, we found that, for detecting Barrett’s esophagus versus healthy, we achieved a sensitivity of 76.2% and a specificity of 82.8%. This falls just short of the ASGE guidelines.^37^ However, for SCC versus healthy, we find >99% sensitivity and 89.6% specificity. This meets the ASGE guidelines and is in agreement with experimental pre-clinical SFDI studies that typically achieve values >90% for both sensitivity and specificity when comparing healthy and cancer tissue.^40^^,^^41^

Next, using our method to analyze the impact of noise on resolution, we find that for absorption the FWHM of our instrument spot size is 4.1 mm versus an estimated 3.7 mm for bench-top systems presented in the literature.^17^ Similarly, for scattering, we find that our spot size is 0.74 versus 0.39 mm for bench-top systems in the literature.^17^ This discrepancy in resolution between scattering and absorption is due to the higher observed noise in absorption. We can therefore conclude that our system should feasibly be able to detect lesions of 4 mm size at the current WD used.

### 3.5 Dual-Wavelength Imaging

Finally, we characterized the performance across the two wavelengths. We found that the recovered optical properties varied by ≤10% when the two wavelengths were measured simultaneously rather than sequentially. This demonstrates the capability of the system to image optical properties at two wavelengths simultaneously with relatively low cross-coupling.

**Fig. 10:**
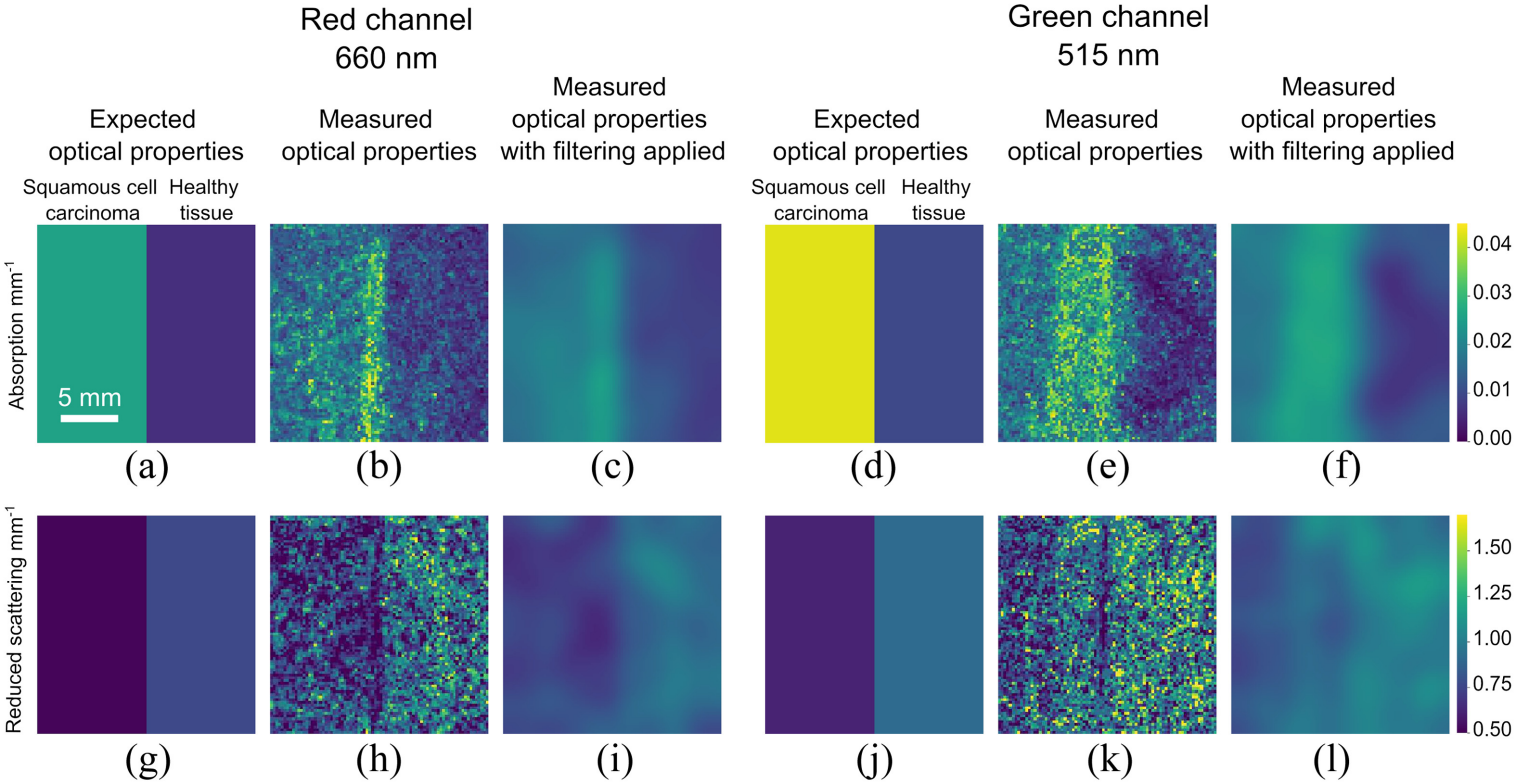
Optical properties measured from dual-wavelength imaging experiment: (a) expected absorption coefficient from red channel; (b) measured absorption coefficient from red channel with (c) filtering applied. (d) Expected absorption coefficient from green channel; (e) measured absorption coefficient from green channel with (f) filtering applied. (g) Expected reduced scattering coefficient from red channel; (h) measured reduced scattering coefficient from red channel with (i) filtering applied. (j) Expected reduced scattering coefficient from green channel; (k) measured reduced scattering coefficient from green channel with (l) filtering applied.

We then imaged two phantoms with different optical properties placed adjacent to one another, one mimicking the optical properties of SCC and the other mimicking the optical properties of healthy esophageal tissue. The results are shown in Figs. 10(a)–10(l). The difference in optical properties is visible from both the red and green channels. The optical properties measured from the red and green channels are not expected to be the same as the phantom properties shift with wavelength.^34^ We expect the phantom optical properties measured from the green channel to be higher than phantom optical properties measured from the red channel.

## 4 Discussion

We have developed an ultra-miniature SFDI system and shown its capability to quantitatively image differences in optical properties of typical gastrointestinal conditions simulated in tissue-mimicking phantoms, providing enhanced contrast. It is sufficiently small to fit in the instrument channel of a standard colonoscope (<3 mm). This work could therefore form the basis of new devices suitable for cost-effective endoscopic deployment for screening of gastrointestinal cancers.

This work has limitations that need further investigation before clinical translation. The first limitation is the choice of wavelengths, which in these experiments was 660 and 515 nm. By evaluating the absorption coefficient at two wavelengths, tissue information such as chromophore concentration can be determined. Oxyhemoglobin (${\mathrm{HbO}}_{2}$) and deoxyhemoglobin (Hb) are important tissue optical properties because they can detect perfusion, which enables differentiation between malignant and benign tumors,^42^ though wavelengths of 670 and 850 nm are more commonly used.^9^ Our system has two constraints that make it challenging to extend to the NIR, e.g., 850 nm. First, the micro camera module has an IR filter that blocks light in this range, but future versions may remove this. Second, the fiber array was designed for 660 nm, and therefore it is very lossy when using a 850 nm laser, with <1% efficiency. In the future, a fiber array could be designed to operate successfully at both 660 and 850 nm: indeed fiber arrays with low-coupling between cores and that operate well into the NIR (1550 nm) are routinely used in telecommunications.^43^

The second limitation is the need for real time operation for clinical application. Our analysis shows that it typically takes between 0.42 and 2.34 s to obtain three suitable frames for SFDI (sufficient contrast with three shifted phases) while the fiber is being manually perturbed, giving an effective SFDI frame rate of at most 2.4 fps. If the system was kept still, it would take longer to acquire the three desired frames, but in this case, artificial phase perturbation could be introduced by means of a phase-shifter (>100  MHz operation), mechanical agitator (e.g., mode scrambler), or spatial light modulator (>50  Hz operation). This would greatly reduce the time required to collect three phases, which could approach ∼30  ms for a 100 fps camera. However, the phase tracking algorithm is currently relatively slow and runs offline (taking several minutes), so it does not allow for real-time operation. This could be addressed by implementing the algorithm on a fast GPU that processes images as they arrive. Alternatively, images with non-optimal phases could be used for sinusoid fitting instead of waiting for three equispaced phases.^44^

The third limitation is image quality, which is somewhat reduced by the non-ideal illumination patterns produced by the fiber array. This causes both noise and some inaccuracies in optical properties. The noise has been partially addressed by varying the spatial frequency but could be further improved by normalizing for laser power, using AI^45^ for noise-reduction reconstruction or building custom LUTs based on non-ideal projection patterns.^32^ The underestimation of absorption at larger absorption levels can be up to 60%, though this too may be reduced by alternative calibration procedures.^39^ This does not appear to significantly impact the quantitative maps of tissue produced and, more importantly, still permits a suitably high theoretical sensitivity and specificity. According to the American Society of Gastrointestinal Endoscope (ASGE) guidelines, a new device requires 90% sensitivity and 80% specificity to be used in lieu of biopsy sampling.^37^ Post-processing analysis of our data suggests that the detection of Barrett’s esophagus may fall just short of this, although this could be improved by binning together pixels at the expense of spatial resolution. However, we show that it is possible to exceed these values when comparing healthy and SCC models, achieving >99% sensitivity and 89.6% specificity. This shows that, even in the presence of visually perceptible noise, absorption and scattering when used in combination remain sufficiently robust to provide maps of SCC. However, we note that our simulated values of sensitivity and specificity are probably higher than what would be expected in a preclinical study as they do not consider other confounding pathologies such as neoplasias or inflammation. The noise in reconstructed absorption and scattering contributes to a reduction in resolution, but even for absorption, which is noisier than scattering, we calculate an achievable smallest feature size of 4 mm. This is substantially smaller than the 6 mm threshold for lesion size in colon polyps used to determine the risk of progression to cancer.

Further miniaturization of the device could look at the use of metasurfaces for polarizers on the fiber tip,^46^ various fiber tip filters to image different reflected wavelengths,^47^ or patterned surfaces to produce a concentric circle illumination pattern required for wide-field imaging inside tubular lumen.

## 5 Conclusion

We have shown the capability of an ultra-miniature (3 mm diameter) SFDI system to detect quantifiable variances in absorption and reduced scattering coefficients in tissue mimicking phantoms with errors of 15% and 6%, respectively, compared to a conventional bench-top SFDI system. Our device has the capability to project two wavelengths simultaneously, enabling extraction of additional properties such as tissue chromophore information. We fabricated tissue-mimicking phantoms simulating typical gastrointestinal condition of SCC adjacent to healthy esophageal tissue, where the absorption coefficient of SCC is much greater than that of healthy tissue and the reduced scattering coefficient is lower. We have shown the capability of our system to image this variation at both one and two wavelengths simultaneously, providing enhanced contrast between the two tissue types. Because of its small size and therefore compatibility with endoscopic deployment, we envisage that this system could be used for cost-effective endoscopic screening of gastrointestinal cancers.

## Data Availability

The data presented in this study are available from the Nottingham Research Data Management Repository, 10.17639/nott.7350.
